# Exact Solution for Viscoelastic Flow in Pipe and Experimental Validation

**DOI:** 10.3390/polym14020334

**Published:** 2022-01-15

**Authors:** Ekaterina Vachagina, Nikolay Dushin, Elvira Kutuzova, Aidar Kadyirov

**Affiliations:** Institute of Power Engineering and Advanced Technologies, FRC Kazan Scientific Center, Russian Academy of Sciences, 420111 Kazan, Russia; vachaginae@mail.ru (E.V.); ndushin@bk.ru (N.D.); elvira.kutuzova@list.ru (E.K.)

**Keywords:** polymer solution, Giesekus, eXtended Pom-Pom, visualization, analytical solution

## Abstract

The development of analytical methods for viscoelastic fluid flows is challenging. Currently, this problem has been solved for particular cases of multimode differential rheological equations of media state (Giesekus, the exponential form of Phan-Tien-Tanner, eXtended Pom-Pom). We propose a parametric method that yields solutions without additional assumptions. The method is based on the parametric representation of the unknown velocity functions and the stress tensor components as a function of coordinate. Experimental flow visualization based on the SIV (smoke image velocimetry) method was carried out to confirm the obtained results. Compared to the Giesekus model, the experimental data are best predicted by the eXtended Pom-Pom model.

## 1. Introduction

Nonlinear differential rheological equations of state are being used increasingly often to describe the rheological properties of viscoelastic fluids and to solve fluid mechanics problems related to polymer melts and solutions. First of all, the features of viscoelastic polymer-based materials behavior include stress-relaxation phenomena, the presence of elastic properties, nonlinear dependence of effective viscosity on shear rate, the occurrence of normal stresses in shear flows, and the ability to swell.

The rheological models based on relaxation equations of state and the structure of polymer molecules, which describe all the above phenomena that occur in flows of viscoelastic polymer materials in the channels of production equipment, are well known in the literature. Analytical solutions can be obtained using simple equations or under rather rough simplifying conditions. As a consequence, most of the studies deal with unimodal viscoelastic rheological models, e.g., Gruz and Pinho [1,2] (Poiseuille-Couette flows of PTT fluids), Oliveira [3] (Fene-P and Giesekus fluid flows in round pipes and flat channels), Schleiniger and Weinacht [4], or a number of works by Oliveira et al. [5] and Coelho et al. [6,7] (isothermal and non-isothermal Fene-P and PTT fluid flows in round pipes and flat channels [5,8]), Hashemabadi [9,10]. Nevertheless, the rheological properties of polymeric melts and concentrated polymer solutions are very often more complex than predicted by such unimodal models, and hence multimode models providing more adequate description are required. In [11], an analytical solution was obtained for a simplified multimode rheological model of a PTT fluid flow. In most cases, calculations of multimode viscoelastic flows are carried out by numerical methods, e.g., [12,13]. The disadvantage of the numerical approach to solution of the considered problems is that for each particular case, it is necessary to perform a full complex of numerical studies, which, due to strong nonlinearity of the considered equations, requires significant computational resources. In contrast, the parametric representation of solution yields distributions of velocities and stresses for a wide variety of problems with any degree of accuracy.

Analytical methods for viscoelastic fluid flows described in [4,5,6] employed simplified rheological models which led to relations connecting the nonzero components of the elastic strain tensor and the shear-rate gradient. Using the solution to the momentum transfer equation in projection onto the direction of a viscoelastic medium motion for such simplified models, the authors [4,5,6] then obtained a correlation between a specific selected unknown function and a transverse coordinate. In this case, the remaining unknown functions are expressed in terms of the selected one using the obtained relations. Our solution method does not have the drawback of using simplified rheological models and can be adapted to an arbitrary number of the rheological model modes. Some experimental studies were carried out to visualize the viscoelastic medium flow in a round pipe to check the obtained results of theoretical studies. The experimental data were compared with the results obtained using the Giesekus model and Extended Pom-Pom.

## 2. Problem Statement

### 2.1. Mathematical Model

A steady laminar isothermal flow of viscoelastic incompressible fluid in around pipes was considered. We assumed that the velocity vector had a single axial velocity component *V_z_*, which is a function of the only variable r of a cylindrical coordinate system r,φ,z with the z-axis directed along the pipe axis. Under the assumptions made, the system of equations for the momentum transfer and continuity in the selected coordinate system can be written as

(1) $$-\frac{\partial P}{\partial z}+\frac{1}{r}\frac{d\left(r\sigma_{rz}\right)}{dr}=0,-\frac{\partial P}{\partial r}+\frac{1}{r}\frac{d\left(r\sigma_{rr}\right)}{dr}-\frac{\sigma_{\varphi \varphi }}{r}=0,-\frac{\partial P}{\partial \varphi }=0,\frac{dV_{z}}{dz}=0$$

where *P* is the pressure; $\sigma_{rr},\;\sigma_{\varphi \varphi },\;\sigma_{rz}$ are physical components of the stress tensor in the cylindrical coordinate system. Let us assume that the liquid adheres to the pipe wall. It follows from (1) that

(2) $$\sigma_{rz}=-\left|C_{0}\right|r/2,\;\partial P/\partial z=C_{0}=const=-\left|C_{0}\right|$$

The rheological equation of state for a multimode viscoelastic fluid can be written as

$$\sigma =\sum_{k=1}^{n}\sigma_{k}+\sigma_{N},\sigma_{N}=2\eta_{N}D$$

where *n* is the total number of modes; σ is the extra stress tensor; $\sigma_{N}$ is the Newtonian part of extra stress tensor; $\eta_{N}$ is the solvent viscosity; $\sigma_{k}$ is the elastic part of extra stress tensor corresponding to each mode.

Two rheological models of viscoelastic behavior were used to determine the elastic components of each mode:

Giesekus model [14]:

(3) $$\sigma_{k}+\lambda_{k}\overset{\nabla }{\sigma_{k}}+\frac{\alpha_{k}\lambda_{k}}{\eta_{k}}\sigma_{k}\cdot \sigma_{k}=2\eta_{k}D,\left(k=1,\ldots ,n\right)$$

Single-equation eXtended Pom-Pom [15] (hereinafter mentioned as eXt. Pom-Pom):

(4) $$f\left(\sigma_{k}\right)\sigma_{k}+\lambda_{k}\left({\overset{\nabla }{\sigma }}_{k}\right)+\frac{\lambda_{k}\alpha_{k}}{\eta_{k}}\left(\sigma_{k}\cdot \sigma_{k}\right)+\frac{\eta_{k}}{\lambda_{k}}\left(f\left(\sigma_{k}\right)-1\right)I=2\eta_{k}D$$

where $f\left(\sigma_{k}\right)=\frac{2}{\varepsilon_{k}}\exp\left[\frac{2\left(\Lambda_{k}-1\right)}{q_{k}}\right]\left(1-\frac{1}{\Lambda_{k}}\right)+\frac{1}{\Lambda_{k}{}^{2}}\left(1-\frac{\alpha_{k}\lambda_{k}{}^{2}tr\left(\sigma_{k}\cdot \sigma_{k}\right)}{3\eta_{k}{}^{2}}\right)$, $\Lambda_{k}=\sqrt{1+\frac{\lambda_{k}tr\sigma_{k}}{3\eta_{k}}}$ is the backbone stretch, $\varepsilon_{k}=\frac{\lambda_{s0k}}{\lambda_{k}}$, k=1,…,n; *k* is the current mode number, $\overset{\nabla }{\sigma }=\frac{d\sigma }{dt}-\sigma \cdot \nabla V^{T}-\nabla V\cdot \sigma$ is the upper convective derivative of the tensor σ; I is the unit tensor; p is the pressure; $D=\left(\nabla V+{\left(\nabla V\right)}^{T}\right)/2$ is the rate of deformation tensor; V is the velocity; $\lambda_{k}$ is the relaxation time; $\eta_{k}$ is the polymeric viscosity; *q_k_* is the number of arms at the backbone extremity of the Pom-Pom molecule; $\alpha_{k}$ and $\varepsilon_{k}$ are the rheological parameters.

### 2.2. Parametric Method

In the present paper, we suggest that a solution method based on the search for an unknown functional dependency $V_{z}\left(r\right)$ should be used in a parametric form. Let us consider a solution method for multimode Giesekus fluid flows in round pipes. For this purpose, we rewrite (3) in a cylindrical coordinate system according to [16]. After some transformations and introduction of the parameter

(5) $$\rho_{k}=\frac{\lambda_{k}}{\eta_{k}}\left|\sigma_{rr(k)}\right|$$

we obtain the following expression for nonzero components of the elastic stress tensor and the shear rate gradient:

(6) $$\sigma_{rz(k)}=-\frac{\eta_{k}}{\lambda_{k}}A_{k},\;\sigma_{zz(k)}=\frac{\eta_{k}\rho_{k}}{\alpha_{k}\lambda_{k}}\left(\frac{2-\alpha_{k}\left(\rho_{k}+1\right)}{\left(1-\rho_{k}\right)}\right),\;\sigma_{rr(k)}=-\frac{\eta_{k}}{\lambda_{k}}\rho_{k}$$

(7) $$\frac{dV_{z}}{dr}=-\frac{1+\rho_{k}\left(1-2\alpha_{k}\right)}{{\left(1-\rho_{k}\right)}^{2}}\frac{A_{k}}{\lambda_{k}}\left(k=1,\ldots ,n\right)$$

Hereinafter $A_{k}=\frac{\sqrt{\rho_{k}\left(1-\alpha_{k}\rho_{k}\right)}}{\sqrt{\alpha_{k}}}$.

Introducing the parameter $\rho_{k}$ into (6), we get

(8) $$\sigma_{rz}=-\frac{\eta_{N}\left(1-2\alpha_{k}\rho_{k}+\rho_{k}\right)}{\lambda_{k}{\left(1-\rho_{k}\right)}^{2}}A_{k}-\sum_{j=1}^{n}\frac{\eta_{j}}{\lambda_{j}}A_{j}\;\left(k=1,\ldots ,n\right)$$

(9) $$\tilde{r}=\frac{2R}{\left|C_{0}\right|}\left(\frac{\eta_{N}\left(1-2\alpha_{k}\rho_{k}+\rho_{k}\right)}{\lambda_{k}{\left(1-\rho_{k}\right)}^{2}}A_{k}+\sum_{j=1}^{n}\frac{\eta_{j}}{\lambda_{j}}A_{j}\right),\;(\tilde{r}=r/R)$$

The remaining components of the stress tensor can be obtained similarly: $\sigma_{zz}$, $\sigma_{rr}$.

The parameter $\rho_{k}$ was introduced for each of the modes; therefore, to obtain the parametric dependences $dV_{z}\left(r\right)/dr$, $V_{z}\left(r\right)$, and $\sigma_{ij}\left(r\right)$ we select the parameter $\rho_{1}$, corresponding to the first mode. The remaining parameters $\rho_{k}\left(k\neq 1\right)$ must be expressed in terms of the main one $\rho_{1}$ using equations (7).

The parametric dependence of the dimensionless shear rate gradient will have the form:

(10) $$\{\begin{array}{c} \frac{dv_{z}\left(\rho_{1}\right)}{d\tilde{r}}=-\frac{1+\rho_{1}\left(1-2\alpha_{1}\right)}{{\left(1-\rho_{1}\right)}^{2}}\frac{A_{1}}{{\tilde{\lambda }}_{1}Wi}\\ \tilde{r}\left(\rho_{1}\right)=\frac{1}{Kr}\left(\frac{{\tilde{\eta }}_{N}\left(1-2\alpha_{1}\rho_{1}+\rho_{1}\right)A_{1}}{{\tilde{\lambda }}_{1}{\left(1-\rho_{1}\right)}^{2}}+\sum_{j=1}^{n}\frac{{\tilde{\eta }}_{j}\sqrt{\rho_{j}\left(\rho_{1}\right)\left(1-\alpha_{j}\rho_{j}\left(\rho_{1}\right)\right)}}{\sqrt{\alpha_{j}}{\tilde{\lambda }}_{j}}\right) \end{array}$$

where $\rho_{j}\left(\rho_{1}\right)$ is determined with any degree of accuracy from (7) using numerical methods, for example, by dichotomy method; ${\tilde{\lambda }}_{j}=\lambda_{j}/\lambda_{a}$, ${\tilde{\eta }}_{j}=\eta_{j}/\eta_{0}$ and ${\tilde{\eta }}_{N}=\eta_{N}/\eta_{0}$ are dimensionless simplexes; $\lambda_{a}=\sum_{i=1}^{n}\lambda_{i}\eta_{i}/\sum_{i=1}^{n}\eta_{i}$ is the average relaxation time; $\eta_{0}=\eta_{N}+\sum_{i=1}^{n}\eta_{i}$ is the maximum possible fluid viscosity; $v_{z}=V_{z}/V_{a}$ is the dimensionless velocity; $V_{a}=Q/\left(\pi R^{2}\right)$ is the average bulk velocity; Q is the fluid flow rate through the cross-section of the circular pipe; *R* is the radius of the circular pipe. Here $Wi=\left(\lambda_{a}R/V_{a}\right)$ is the Weissenberg number, Kr=Wi⋅Eu⋅Re0⋅Γ is the dimensionless complex; $Eu=\left|C_{0}l\right|/\left(\rho V_{a}{}^{2}\right)$ is the Euler number; Re0=(ρVa(2R))/η0 is the Reynolds number; l is the pipe length; $\Delta P=\left|C_{0}l\right|$ is the pressure drop in a round pipe across the length l.

To obtain the dependence of the dimensionless velocity $v_{z}$ on the dimensionless variable in a parametric form, it is necessary to use the condition

(11) $$\int_{0}^{1}v_{z}\tilde{r}d\tilde{r}=\frac{1}{2}$$

The parametric dependence of the axial component of the dimensionless velocity on the dimensionless coordinate can be obtained from (10) by integrating these relations

(12) $$\{\begin{array}{c} \begin{array}{l} v_{z}\left(\rho_{1}\right)=\frac{{\tilde{\eta }}_{N}}{2KrWi{\tilde{\lambda }}_{1}{}^{2}\alpha_{1}}\frac{{\left(1+\left(1-2\alpha_{1}\right)\rho_{1}^{w}\right)}^{2}\rho_{1}^{w}\left(1-\alpha_{1}\rho_{1}^{w}\right)}{{\left(1-\rho_{1}^{w}\right)}^{4}}\\ -\frac{{\tilde{\eta }}_{N}}{2KrWi{\tilde{\lambda }}_{1}{}^{2}\alpha_{1}}\frac{{\left(1+\left(1-2\alpha_{1}\right)\rho_{1}\right)}^{2}\rho_{1}\left(1-\alpha_{1}\rho_{1}\right)}{{\left(1-\rho_{1}\right)}^{4}}+\frac{1}{2KrWi}\sum_{j=1}^{n}\frac{{\tilde{\eta }}_{j}}{{\tilde{\lambda }}_{j}{}^{2}\alpha_{j}}B\left(\rho_{j}\right) \end{array}\\ \tilde{r}\left(\rho_{1}\right)=\frac{1}{Kr}\left(\frac{{\tilde{\eta }}_{N}\left(1-2\alpha_{1}\rho_{1}+\rho_{1}\right)A_{1}}{\lambda_{1}{\left(1-\rho_{1}\right)}^{2}}+\sum_{j=1}^{n}\frac{{\tilde{\eta }}_{j}\sqrt{\rho_{j}\left(\rho_{1}\right)\left(1-\alpha_{j}\rho_{j}\left(\rho_{1}\right)\right)}}{\sqrt{\alpha_{j}}\lambda_{j}}\right) \end{array}$$

where $B\left(\rho_{j}\right)=\int_{\rho_{j}}^{\rho_{1}^{w}}\frac{\left(1+\left(1-2\alpha_{j}\right)\rho_{j}\right)\left(1-2\alpha_{j}\rho_{j}\right)}{{\left(1-\rho_{j}\right)}^{2}}d\rho_{j}$ is calculated analytically; $\rho_{1}^{w}$ is the parameter value on the pipe wall.

The direct use of analytical expressions (12) is hampered by the fact that there is a functional dependence between the dimensionless complexes Wi and Kr, which can be determined using (11).

Similarly, a parametric problem solution for multimode fluid flows can be obtained using eXt. Pom-Pom can be obtained. In this case, it is convenient to use the following expression as a parameter ($\rho_{k}$)

(13) $$\sigma_{\varphi \varphi (k)}=\frac{{\tilde{\eta }}_{k}}{{\tilde{\lambda }}_{k}}\frac{\eta_{0}}{\lambda_{a}r^{2}}\left(-\rho_{k}\right),\;{\tilde{\eta }}_{k}=\eta_{k}/\eta_{0},\;{\tilde{\lambda }}_{k}=\lambda_{k}/\lambda_{a}.\;(0\leq \rho_{k}\leq 1)$$

Then

(14) $$\left\{ \begin{array}{l} v_{z}\left(\rho_{1}\right)=\int_{\rho_{1}}^{\rho_{1}^{w}}\overset{}{{\overset{\cdot }{\gamma }}_{k}\left(\rho_{1}\right)\left(d\tilde{r}/d\rho_{1}\right)/{\tilde{\lambda }}_{k}}d\rho_{1}\\ \tilde{r}\left(\rho_{1}\right)=-\left(\frac{{\tilde{\eta }}_{N}}{{\tilde{\lambda }}_{m}}{\overset{\cdot }{\gamma }}_{m}\left(\rho_{m}\right)+\sum_{k=1}^{n}\frac{{\tilde{\eta }}_{k}}{{\tilde{\lambda }}_{k}}\frac{\lambda_{k}}{\eta_{k}}\sigma_{rz(k)}\left(\rho_{k}\right)\right){\left(Kr\right)}^{-1} \end{array}\right.$$

where dependences $\rho_{k}\left(\rho_{1}\right)$ can be obtained from the definition of the shear rate gradient

(15) $$\overset{\cdot }{\gamma }=\frac{dv_{z}}{d\tilde{r}}=\frac{{\overset{\cdot }{\gamma }}_{1}}{Wi{\tilde{\lambda }}_{1}}=\ldots =\frac{{\overset{\cdot }{\gamma }}_{n}}{Wi{\tilde{\lambda }}_{n}}$$

A more detailed description of the method is provided in [17].

## 3. Materials and Methods

### 3.1. Experimental Setup

**Figure 1:**
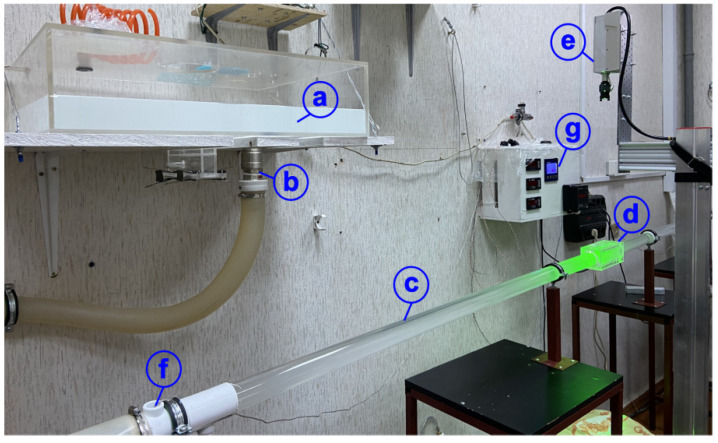
Photo of the test bench. (**a**) tank with polymer solution in distilled water; (**b**) valve; (**c**) Plexiglass pipe; (**d**) rectangular duct; (**e**) laser; (**f**) resistance thermocouple; (**g**) temperature monitor.

Experimental studies to investigate the structure of the viscoelastic fluid flow were carried out on a test bench (FRC KazSC of RAS, Kazan, Russia) shown in Figure 1. The test sections were made of transparent plexiglass with the diameter D1 = 39 (inner)/D1 = 45 (outer) mm (Figure 1c). The inlet and outlet sections had the length of 43 D_1_ sufficient to exclude the inflow and outflow effects. The test section was placed in a rectangular duct (Figure 1d) to compensate for optical distortions. The space between the test section and the duct was filled with a fluid with a refractive index close to that of the test section and the duct. The actual fluid temperature was controlled using a resistance temperature device DTS034-RT100.A3.25/1.5 (OVEN, Moscow, Russia) with the uncertainty of 0.1 °C (Figure 1f,g). The fluid temperature was kept constant using a KENTATSU (KENTATSU DENKI, Guangdong, China) air conditioner installed in the room.

### 3.2. Visualization

**Figure 2:**
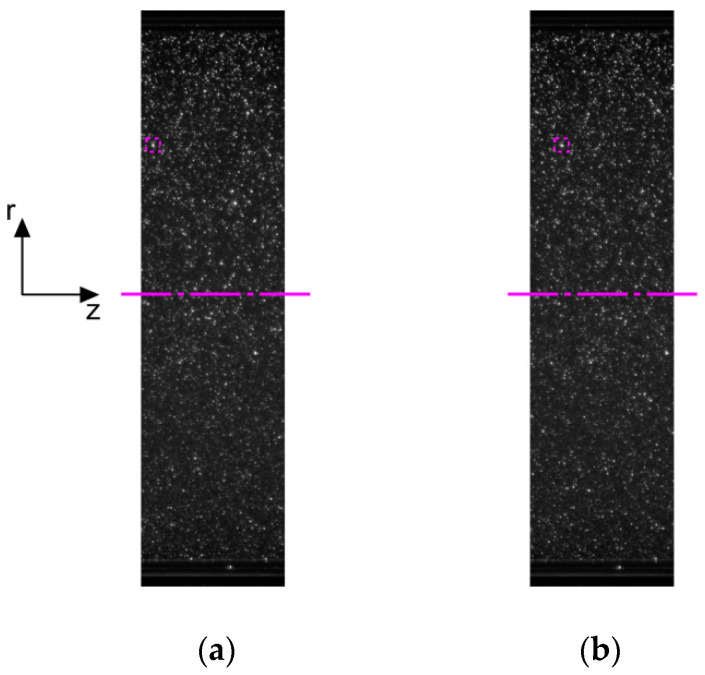
The tracer’s motion at different time: (**a**) $t=t_{0}$; (**b**) $t=t_{0}+\Delta t$.

Velocity profiles were estimated using the Smoke Image Velocimetry (SIV, Kazan, Russia) method developed at our institute. It is an optical measurement method. Articles [18,19] give a detailed description of the SIV method, estimation of its accuracy, and applicability to the measurement of various flow characteristics. The SIV method was chosen due to the peculiarities of the image-processing algorithm. In particular, the SIV method requires neither uniform seeding of the flow with tracers nor smoothing the maximum of the cross-correlation function when refining the displacement of tracers with subpixel accuracy. These aspects simplify the preparation of the working fluid and increase the measurement accuracy. Figure 2 illustrates the tracer’s motion at different times. For example, the motion of one particle is highlighted with a red rectangle. The detailed description of the SIV method is presented in [18,19]. The used frequency value was sufficient to fulfill the condition for the permissible displacement of the tracer images with respect to the size of the compared interrogation windows at the highest flow rates and provided the convenience of a virtual decrease in the shooting frequency at lower flow rates. The virtual decrease in the shooting frequency was used to increase the displacement of tracers in the compared pairs of frames to the optimal values from the standpoint of measurement accuracy (about half the longitudinal size of the interrogation window). The size of the compared interrogation windows in all cases was 28 × 20 pix^2^ and was selected based on the patterns of the distribution of tracers in the flow visualization frames. The scaling factor was 24.4 pix/mm. The spacing between grid nodes was 20 pix (0.82 mm). The shooting time and the number of captured frames were sufficient for time averaging of the data. For most of the investigated modes, 1000 pairs of frames were compared.

The laser light sheet in the experiments was created by a continuous diode laser SSP-ST-532-NB-LED (Changchun New Industries Optoelectronics Tech. Co. Ltd, Changchun, China) (Figure 1e). The laser light sheet was no more than 0.6 mm thick. Filming was carried out with a Phantom Miro C110 digital video camera (Vision Research, Inc., Wayne, New Jersey, USA) with a frequency of 60 fps in steady flow regimes. The image processing procedure included elimination of static objects and noise filtering. The procedures for searching and filtering erroneous velocity vectors were applied to the primary quantitative data. The average number of erroneous vectors in the entire volume of the obtained data was no more than 0.56%.

### 3.3. Polymer Solution Preparation

An aqueous solution of polyacrylamide with polyamide particles (average diameter 20 μm) was used as a viscoelastic liquid. The influence of gravity and inertia forces on the position of tracers relative to the carrier medium during the measurements was negligible. The fluid was prepared in the following order. Polyamide particles with a concentration of 0.013% of the total mass were added to warm distilled water and slightly (manually) mixed. Then, the mixing process was carried out using an overhead stirrer Heidolph Hei-TORQUE Value 100 (Heidolph Instruments GmbH & Co. KG, Schwabach, Germany) with a ViscoJet stirrer (Heidolph Instruments GmbH & Co. KG, Schwabach, Germany). After two minutes of mixing, the polyacrylamide powder was gradually added. It took 15 min to mix 700 mL volume. The uncertainty in weighing of distilled water, polyacrylamide powder, and polyamide particles was less than 0.1%. Prior to the experiment, the finished liquid was stored in 5 L canisters in a dark cabinet.

### 3.4. Rheological Measurements

The viscosity curve, as well as dynamic moduli of two concentration of polymer solutions (2500 and 7500 ppm weight), were measured using MCR 102 rheometer (Anton Paar, Graz, Austria) equipped with a Peltier (H-PTD200) (Anton Paar, Graz, Austria) temperature control system with an accuracy of 0.01 °C; parallel-plate geometry was employed with a plate diameter of 50 mm with 1 mm gap.

## 4. Results and Discussion

**Figure 3:**
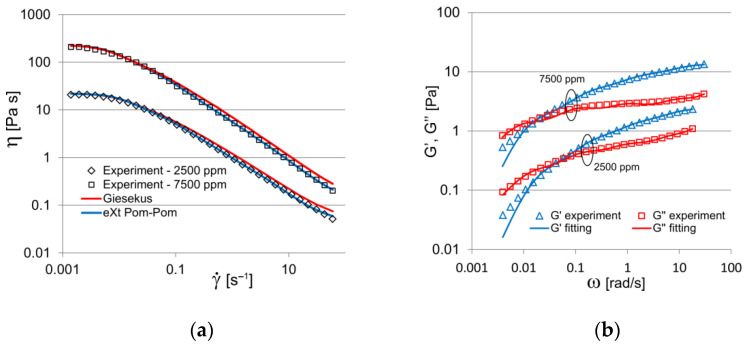
Viscosity curve (**a**) and dynamic moduli (**b**); experiment and fitting with four-mode Giesekus and eXt. Pom-Pom models (2500 ppm at *T* = 22.8 °C and 7500 ppm at *T* = 20.0 °C).

Figure 3 shows the experimental viscosity curve and dynamic moduli of tested samples of polymer solutions (the temperature value was taken from experimental studies of flow visualization). Intermediate calculations showed that four modes are sufficient to approximate the dynamic modulus curves and the viscosity curve, which is consistent with the literature [11] (Figure 3). The literature data [20] were used to find the linear spectrum of the relaxation time for the four-modal rheological equations of state of a viscoelastic medium. The search for a set of nonlinear parameters of rheological models (3) and (4) is based on the flow curve approximation; e.g., for the Giesekus model, a detailed algorithm was presented in our earlier published work [21]. For the reader’s convenience, Table 1 presents the parameters of the Giesekus and eXt. Pom-Pom models that we found, which characterize the rheological behavior of the tested samples (2500 and 7500 ppm). From the figure, we can see that the eXt. Pom-Pom model, in comparison with the Giesekus model, best approximates the experimental viscosity curves for both concentrations, especially in the shear rate range $0.1<\dot{\gamma }<60$. A slight deviation in the storage modulus approximation in the interval ω<0.02 was caused by the use of four modes; however, as is shown below, this deviation does not significantly affect the final result.

Since the specific values of the average bulk velocity ($V_{a}$) used in flow visualization were calculated during post-processing by integrating the velocity profile over the pipe section according to the formula

(16) $$V_{a}=2\int_{0}^{R}V_{z}rdr/R^{2}$$

then analytical solutions were obtained using the initial experimental data.

For 2500 ppm, we visualized four variants of the flow with the values *Va* = 1.866; 4.579; 7.511; 9.588 [mm/s], which corresponded to the following Weissenberg numbers: *Wi* = 4.80; *Wi* = 11.76; *Wi* = 19.29; *Wi* = 24.63. For 7500 ppm, two variants with the values *Va* = 0.993; 1.561 [mm/s] were investigated, which corresponded to the following *Wi* = 4.29; 6.74.

**Figure 4:**
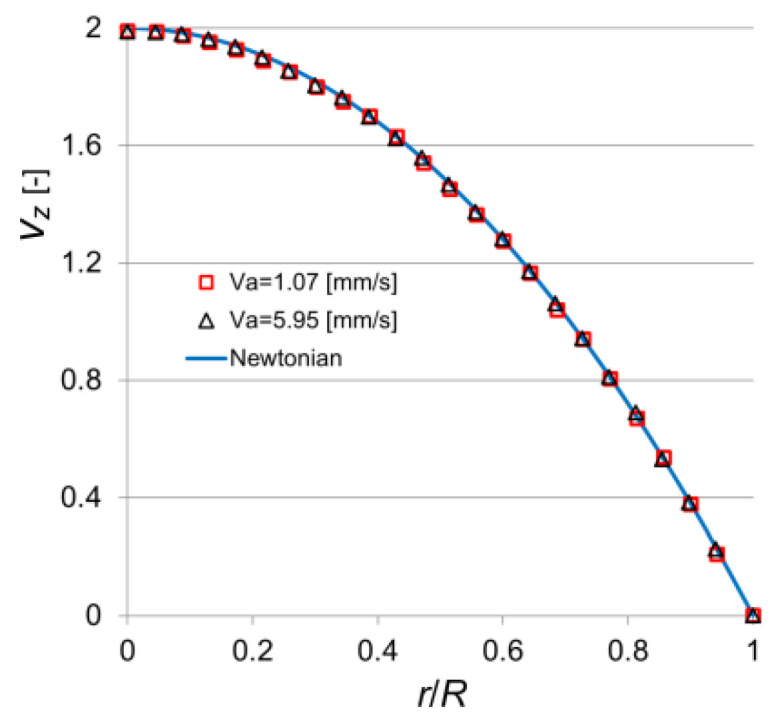
Dimensionless axial velocity profiles in the pipe for various *v*_a_ [mm/s] (Newtonian flow, the mixture of 40% glycerol and 60% propylene glycol with dynamic viscosity *μ* = 166 × 10^−3^ Pa·s; *T* = 22.6 °C).

The visualization method was preliminarily tested on the flow of 40% glycerol and 60% propylene glycol (Figure 4). Good agreement of the experimental data with the parabolic velocity profile characterizing the laminar flow of Newtonian fluid in a round pipe was obtained. The relative uncertainty did not exceed 0.6%.

**Figure 5:**
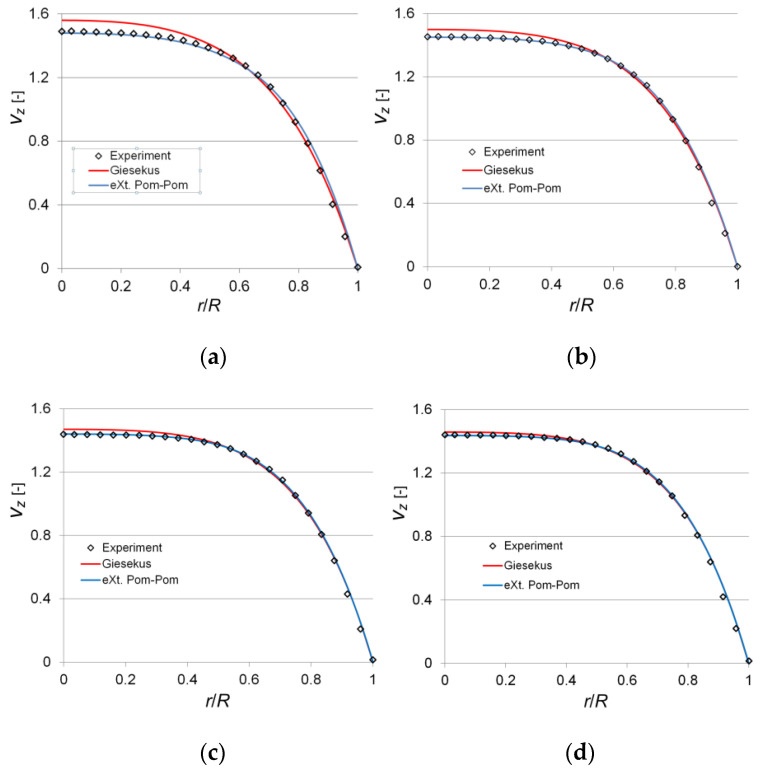
Dimensionless axial velocity profiles in the pipe for various *Wi* ($\lambda_{a}=50.087{\;s}^{-1}$ ): (**a**) *Wi* = 4.80; (**b**) *Wi* = 11.76; (**c**) *Wi* = 19.29; (**d**) *Wi* = 24.63.

**Figure 6:**
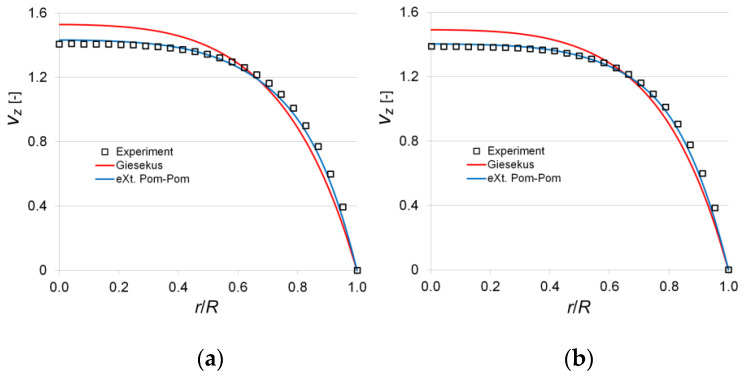
Dimensionless axial velocity profiles in the pipe for various *Wi* ($\lambda_{a}=84.25{\;s}^{-1}$): (**a**) *Wi* = 4.29; (**b**) *Wi* = 6.74.

Figure 5 and Figure 6 show the profiles of dimensionless axial velocity in the longitudinal section of the channel, which characterize the laminar flow of tested polymer solutions in the circular pipe. The illustration of the obtained data shows that the experimental data are best predicted using the eXt. Pom-Pom model, while the relative uncertainty consequently does not exceed 0.9 and 0.96% for 2500 and 7500 ppm. For the Giesekus model, there is a tendency to overestimate the axial velocity values on the channel axis with a decrease in the Weissenberg number. For 2500 ppm, the relative uncertainty on the channel axis does not exceed 1.2% at *Wi* = 24.63 and it is 4.66% at *Wi* = 4.80. For 7500 ppm the discrepancy is more pronounced, so the relative uncertainty equals 8.77% at *Wi* = 4.29 and 7.49% at *Wi* = 6.74. Additional calculation was performed for a particular case, i.e., *Wi*→0 (tending to Newtonian flow), to check the correctness of the developed method. At *Wi* = 0.01, the calculated velocity profiles predicted by both the Giesekus and eXt. Pom-Pom models coincided with the parabolic profile. The latter indicates that even for the simplest case of viscoelastic fluid flow in a round pipe, the Giesekus model overestimates the value of axial velocity in the central region of the channel in the range of Weissenberg numbers 0.1 < *Wi* < 25. The obtained results agree with the results of other authors who investigated more complex geometries [22].

## 5. Conclusions

Our proposed parametric method for multimode Giesekus fluid flows remains stable in a wide range of Weissenberg numbers, but the velocity profiles predicted by this model are slightly higher than the experimental data in the central region of the channel. The best agreement with the experimental data was obtained using the eXt. Pom-Pom model for example of pipe flows of aqueous polyacrylamide solutions with 2500 and 7500 ppm concentrations. The SIV method used to visualize turbulent flows demonstrated its efficiency for visualization of viscoelastic fluid flows and can be successfully applied to the analysis of more complex flows.

## Figures and Tables

**Table 1:** Parameters of Giesekus and eXt. Pom-Pom models (2500 ppm at *T* = 22.8 °C, 7500 ppm at *T* = 20.0 °C).

				Giesekus	eXt. Pom-Pom		
Concentration [ppm]	$\lambda_{i}\;[s]$	$\eta_{i}\;[\mathrm{Pa}\cdot s]$	$\eta_{N}\;[\mathrm{Pa}\cdot s]$	$\alpha_{i}$	$q_{i}$	$\varepsilon_{k}$	$\alpha_{i}$
2500	0.1406	0.1253	0.0411	0.495	1	0.1	0.9
	0.991	0.71		0.495	2	0.4	0.5
	7.1911	4.064		0.495	1	0.1	0.7
	62.6357	17.1691		0.495	1	0.1	0.6
7500	0.1106	0.4699	0.0962	0.495	1	0.1	0.6
	1.0032	3.8742		0.495	2	0.4	0.1
	9.0587	31.7676		0.495	1	0.1	0.6
	98.5914	191.802		0.495	1	0.1	0.4

## Data Availability

The data presented in this study are available on request from the corresponding author.
